# A multistakeholder approach to innovations in NAFLD care

**DOI:** 10.1038/s43856-022-00228-y

**Published:** 2023-01-03

**Authors:** Jörn M. Schattenberg, Alina M. Allen, Helen Jarvis, Shira Zelber-Sagi, Ken Cusi, John F. Dillon, Cyrielle Caussy, Sven M. Francque, Zobair Younossi, Naim Alkhouri, Jeffrey V. Lazarus

**Affiliations:** 1 Metabolic Liver Research Program, I. Department of Medicine, University Medical Center, Mainz, Germany; 2 Division of Gastroenterology and Hepatology, Department of Internal Medicine, Mayo Clinic, Rochester, MN USA; 3 Department of Primary Care, Population Health Sciences Institute, Newcastle University, Newcastle upon Tyne, United Kingdom; 4 School of Public Health, University of Haifa, Haifa, Israel; 5 Department of Gastroenterology, Tel-Aviv Medical Center, Tel-Aviv, Israel; 6 Division of Endocrinology, Diabetes and Metabolism, University of Florida, Gainesville, FL USA; 7 Division of Molecular and Clinical Medicine, School of Medicine, University of Dundee, Ninewells Hospital, Dundee, United Kingdom; 8 Univ Lyon, CarMen Laboratory, INSERM, INRA, INSA Lyon, Université Claude Bernard Lyon, Pierre-Bénite, France; 9 Hospices Civils de Lyon, Département Endocrinologie, Diabète et Nutrition, Hôpital Lyon Sud, Pierre-Bénite, France; 10 Department of Gastroenterology Hepatology, Antwerp University Hospital, Edegem, Belgium; 11 InflaMed Centre of Excellence, Laboratory for Experimental Medicine and Paediatrics, Translational Sciences in Inflammation and Immunology, Faculty of Medicine and Health Sciences, University of Antwerp, Wilrijk, Belgium; 12 Center for Liver Disease, Inova Medicine, Falls Church, VA USA; 13 Fatty Liver Program, Arizona Liver Health, Phoenix, AZ USA; 14 Barcelona Institute for Global Health (ISGlobal), Hospital Clínic, University of Barcelona, Barcelona, Spain; 15 CUNY Graduate School of Public Health and Health Policy (CUNY SPH), New York, NY USA; 16 Faculty of Medicine and Health Sciences, University of Barcelona, Barcelona, Spain

**Keywords:** Non-alcoholic fatty liver disease, Non-alcoholic steatohepatitis, Health services

## Abstract

Schattenberg et al. outline discussions from a recent workshop on NAFLD care and advocate for a multidisciplinary approach to managing this complex and multifactorial disease. The authors highlight gaps in current models of care and make recommendations on optimising a multistakeholder approach in steatotic liver diseases.

## Teaser

Non-alcoholic fatty liver disease (NAFLD) is highly prevalent globally and requires multidisciplinary care. Here, we report key findings of a NAFLD care workshop, address knowledge gaps and highlight a path to optimise healthcare resource use, to improve outcomes in patients with steatotic liver disease.

NAFLD, the most common chronic liver disease, affects around 33% of adults worldwide^1^. Given the growing global prevalence of obesity and type 2 diabetes mellitus (T2DM), and their association with NAFLD, the burden of this liver condition and its socio-economic costs are only expected to grow^2^.

NAFLD encompasses a disease spectrum that is defined on the basis of liver histology with fatty infiltration of liver tissue and gradual progression towards chronic inflammation (non-alcoholic steatohepatitis, NASH), fibrosis, cirrhosis and hepatocellular carcinoma (HCC)^3^. There are no approved therapies for NASH, but a growing evidence base supports the effectiveness of non-pharmacological interventions in halting progression or causing remission, highlighting the importance of early diagnosis and clinical management. Additionally, drugs licenced for cardiovascular disease (CVD), like statins, or T2DM, such as glucagon-like peptide 1 (GLP-1) receptor agonists and sodium-glucose cotransporter-2 inhibitors, hold early promise for NAFLD patients^4^.

However, NAFLD and NASH, along with other steatotic (fatty) liver diseases, are largely underdiagnosed worldwide and epidemiological data are scarce^5^, limiting the provision of good practice care and implementation of national preparedness plans. Furthermore, at present, liver biopsy is the most reliable method for diagnosing fibrosis and steatohepatitis, which is limited by cost, sampling error and procedure-related morbidity and mortality^3^, hindering clinical trials and drug development programmes. Several non-invasive tests (NITs), including serum and genetic biomarkers and imaging modalities, have been proposed as options for diagnosing NAFLD and NASH^6^.

NAFLD typically develops in the context of metabolic syndrome (MetS), which includes obesity, T2DM, dyslipidaemia and hypertension^3^. Comprehensive care therefore requires a multidisciplinary and multistakeholder approach, to address the range of comorbidities affecting patients with NAFLD. Recent analyses have shown that a wide range of NAFLD models of care (MoCs) exist, varying in most aspects such as in who provides care, what care is provided considering the disease stage, which healthcare settings care is provided in and to what extent care is coordinated across the healthcare system^7^. Importantly, the availability of auxiliary healthcare services, such as peer-to-peer patient programmes, physician assistants or nurse practitioners, differs widely across healthcare systems.

National preparedness plans are deficient globally, indicating systemic gaps in delivering NAFLD care^8^. While adequate policies and civil society engagement, guidelines^9^, epidemiological data and care management are pivotal to the provision of good care, these are suboptimal to varying degrees in different countries, underscoring a need for dialogue among all relevant stakeholders, to prompt the integration of NAFLD into national public health agendas.

Building on a global public health and NAFLD consensus statement^10^, in May 2022, a multidisciplinary group of some 100 stakeholders met in Barcelona, Spain, and online, to exchange their experience on public health approaches to NAFLD and NASH. The discussions focused on diverse MoCs that currently exist or should be developed, in order to set out priorities for healthcare providers and policymakers, to optimise the diagnosis and management of NAFLD. This unique programme provided an interactive platform for primary care physicians, hepatologists, endocrinologists, nutritionists, public health experts, nurses and patients. Here, we summarise the discussions from this meeting.

## The fatal triple

**Fig. 1:**
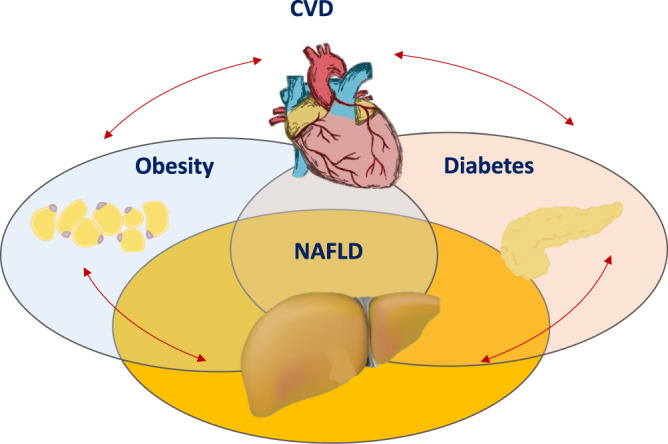
The fatal triple association between obesity, T2DM and CVD, and NAFLD. The conditions are closely associated but without a complete overlap: the estimated prevalence of obesity among T2DM is approximately 80% in the US and the estimated prevalence of obesity among patients with NAFLD is 51% in Europe and the US. The prevalence of NAFLD among T2DM is approximately 55%, whereas the prevalence of NAFLD among patients with obesity varies from 50 to 90%, depending on the severity of obesity. The potential impact of NAFLD on CVD remains controversial, with several mechanisms explaining how NASH can impact the cardiovascular system; however, the independent contribution of NAFLD to the development of CVD is difficult to disentangle from the impact of other factors that are well-established risk factors for CVD. Given these interconnections, a personalised multidisciplinary approach is needed for the optimal care of patients with NAFLD. CVD cardiovascular disease, NAFLD non-alcoholic fatty liver disease, NASH non-alcoholic steatohepatitis, T2DM type 2 diabetes mellitus.

Obesity, T2DM and CVD are closely linked with and impact each other and NAFLD. However, each of these metabolic diseases also has its own unique features, resulting in several sub-types of patients that require a personalised, multidisciplinary approach (Fig. 1). Obesity is a major risk factor for NAFLD, and exposure to obesity increases the risk of HCC^11^. NAFLD increases the risk of developing T2DM, whereas improvement of hepatic steatosis, or fat in the liver, decreases the incidence of T2DM^12^. Furthermore, approximately 55% of patients with T2DM have NAFLD^13^. The presence of NAFLD in patients with T2DM hampers glycaemic control, requiring the use of multiple anti-diabetic therapies and increasing the risk of both micro- and macro-vascular complications^14^. The potential impact of NAFLD on CVD remains controversial. Several mechanisms can explain how NASH can impact the cardiovascular system, offering biological plausibility^15^. However, an independent contribution of NAFLD to the development of CVD is difficult to disentangle from the impact of other factors that are well-established risk factors for clinical CVD events, such as obesity, T2DM, dyslipidaemia and hypertension^3^. The majority of drugs used to prevent or treat CVD, e.g., statins, aspirin and other anti-aggregant drugs, have benefit on the risk factors for NAFLD^15^, but therapies to treat NASH directly are required. Public health measures to address NAFLD include raising awareness and education, in addition to close collaboration between hepatologists, diabetologists, nutritionists and cardiologists, in care provision.

## Screening and risk stratification

People admitted to hospital with decompensated cirrhosis, characterised by liver failure and/or liver-related issues like ascites or jaundice^9^, have a 25% chance of 60-day mortality; approximately 70% of new cirrhosis diagnoses are made on such an admission, making a case for the importance of early detection. Detection strategies based on case finding should focus on risk factors such as MetS features, like T2DM and obesity, or routinely performed liver function tests, and aim to improve the efficiency of existing patient interactions (e.g., automated laboratory measurement analysis to provide decision support to clinicians)^16^. The key to the success of such strategies is risk stratification by focusing on fibrosis markers, as the amount and pattern of fibrosis are indicators of disease progression^9^.

**Fig. 2:**
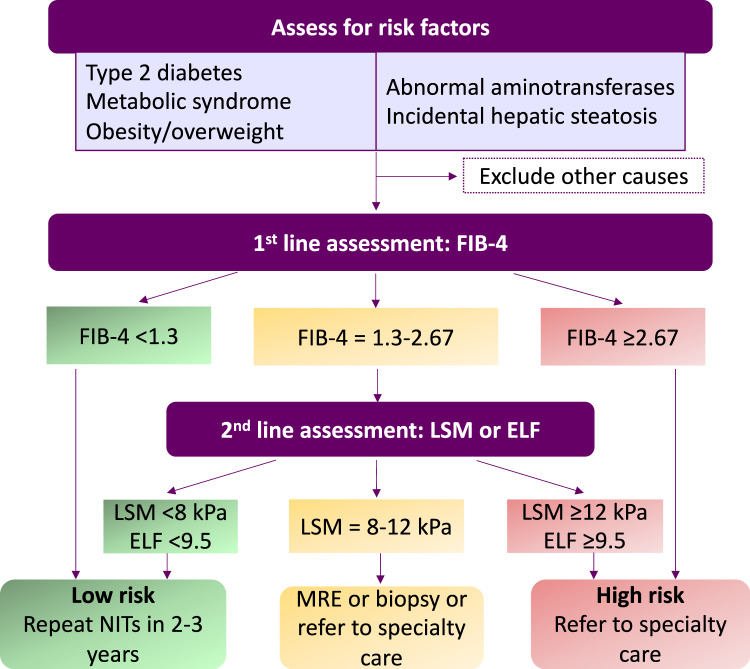
Sequential risk stratification of patients with hepatic steatosis or metabolic risk factors. The sequential use of NITs aiming at the identification of patients with advanced liver disease, histologically defined as stages F2 and F3, allows in a first step to rule-out cases with advanced disease using the FIB-4 score. The presence of metabolic risk factors informs about the at-risk population. This will be crucial to enable the assignation of resources to the group with the highest risk of a detrimental outcome. Patients in the indeterminate category will need to be further evaluated by using a more refined second line test, including LSM or ELF. Patients in the high-risk category should be referred to specialists for further management. Based on the resources and availability of NITs, one-step testing strategies could be used in the future. ELF Enhanced Liver Fibrosis, LSM liver stiffness measurement, MRE magnetic resonance elastography, NIT non-invasive test.

Both the European Association for the Study of the Liver and the American Gastroenterological Association recommend a two-tiered approach to risk stratification in those suspected or incidentally found to have hepatic steatosis on imaging, using sequential NITs (Fig. 2)^17,18^. Although seemingly straightforward and consistent, the success of clinical pathways depends on the level of awareness and action in primary care and by other non-hepatology physicians, who must recognise risk factors, order liver enzyme tests, calculate and interpret NIT results and refer patients to specialists, when appropriate. Referral according to Fig. 2 will help to identify patients with end-stage liver disease and offer care in the context of clinical trials. Repeated testing over years can help decrease false negative rates in NAFLD which is, in general, slow progressing. Moving into the arena of precision medicine and supported by technological progress, risk prediction in the future will likely abandon indirect surrogate scores and incorporate the increasing knowledge on genetics and epigenetic changes, to benefit patients with advanced liver disease as a result of NAFLD. These caveats underscore the critical importance of developing and disseminating risk stratification approaches between multiple disciplines and providing robust data demonstrating improvement in patient outcomes in real-world settings.

## NAFLD management in different settings

As NAFLD is a chronic multisystem disease, its prevention, detection and management require a multidisciplinary approach which should include primary care, hepatology, nutritionists, endocrinology, obesity medicine, bariatric surgery/endoscopy, psychology and exercise physiology^19^ (Box 1). More studies are needed, but a multidisciplinary metabolic hepatology clinic approach has already proven to be effective, as patients with NAFLD experienced reductions in liver enzyme and stiffness levels and cardio-metabolic parameters like cholesterol, glucose and weight^20^. At present, lifestyle interventions are the mainstay of available treatment. Comprehensive MoCs need to incorporate such interventions and integrate and define the roles of primary, secondary and tertiary care throughout the patient’s prevention, detection and management journey^7^.

With a focus on holistic, comprehensive care and secondary prevention, primary care should be ideally placed to find and risk stratify people with NAFLD, while recognising competing workload priorities, positioning NAFLD in the context of existing chronic disease management in different primary healthcare settings and reimbursement schemes and emphasising the need for integrated, centrally approved frameworks.

NAFLD care in the community needs to be part of a wider metabolic multimorbidity management approach, recognising the key role of the primary care nursing team and allied professionals with expertise in preventive hepatology and providing long-term nutrition and lifestyle interventions^9^. To drive change and optimise outcomes, ideal MoCs would include peer support and a role for a NAFLD/metabolic nurse specialist, to closely monitor disease management.

A crucial trigger for successful nutritional treatment is the active support of physicians. There is a vital role for healthcare providers as educators in explaining the importance of treating NAFLD (including in the broader context of T2DM, CVD and cancer prevention) and its potential to regress, teaching healthy eating skills, enhancing confidence in the benefits of diet, discussing potential barriers (e.g., life stressors and the obesogenic environment) and co-creating solutions^21^. Web-based interventions may be more inclusive and evidence indicates that web-based treatment tools are beneficial but dropout rates may be high and these may be more accessible to younger patients^22^. Further studies that integrate available technology with health applications are required.

Finally, although weight loss through traditional lifestyle modifications has been associated with the resolution of NASH and fibrosis regression^23^, only a minority of patients can lose and maintain weight loss. Bariatric surgery leads to significant weight loss and resolution of NASH in the majority of patients and, more importantly, has been shown to reduce both major adverse cardiac events and liver outcomes^24^. Non-surgical options include anti-obesity medications (AOMs), to support patients with NAFLD achieve and sustain their weight loss goals. Oral AOMs have low adherence rates, insufficient data on the histologic improvement of NASH/fibrosis and inconclusive data on their effects on the liver^25^, making them less than ideal for achieving the desired weight loss in fibrotic NASH. Emerging evidence indicates that GLP-1 receptor agonists^26^ can improve steatohepatitis, but it will remain to be determined if any compound meets the regulatory barrier of conditional approval. Whether through lifestyle, pharmaceuticals, surgery or a combination of these, weight management strategies should involve multidisciplinary input and be tailored to each patient.

Box 1 Recommendations to enable and optimise a multidisciplinary approach in NAFLD care

- Evaluate the patient benefit and cost-effectiveness of multidisciplinary models of care based on non-invasive tests, which should be adopted by first-line providers, such as primary care physicians and endocrinologists, and monitor their use.
- Implement *context-specific* (prevention, screening, risk-stratification, treatment) and *resource-specific* (for low-, middle- and high-income settings) models of care and always report their effectiveness.
- Develop, provide and monitor clear guidance, endorsed by the relevant professional societies, regarding preventive hepatology and specific criteria for referral to specialty care such as hepatology, cardiology, endocrinology, obesity specialists, bariatric surgeons and exercise physiologists.
- Delivery models must overcome the time-constraints of primary care physicians and other doctors by decreasing the human burden (by using e.g., automated methods of screening and risk-stratification, multidisciplinary ‘metabolic’ clinics or training, virtual care, nurse coordinators across disciplines, web-based applications for longitudinal care, virtual assistants).
- Generate evidence to provide policy-makers the basis to recognise NAFLD as significant public health threat and allocate the appropriate resources to address it.
- The World Health Organization should integrate all types of steatotic liver disease into non-communicable disease technical guidance, action plans and strategies, setting out how to best implement a multidisciplinary approach.

NAFLD non-alcoholic fatty liver disease.

## Conclusions

Steatotic liver disease is a complex, multisystem disease that requires a multidisciplinary approach to prevention, diagnosis, treatment and care. Such an approach must be bottom-up, with key actors in the health system collaborating and developing efficient MoCs, and top-down, guided by national and global strategies. While no NAFLD- or NASH-specific treatment exists, structured lifestyle interventions and medications for related conditions can have positive effects, and many different stakeholders can and should play a role in improving health outcomes in patients with fatty liver disease.

## References

[1] Riazi K (2022). The prevalence and incidence of NAFLD worldwide: a systematic review and meta-analysis. Lancet. Gastroenterol. Hepatol..

[2] Schattenberg JM (2021). Disease burden and economic impact of diagnosed non-alcoholic steatohepatitis in five European countries in 2018: a cost-of-illness analysis. Liver. Int..

[3] Chalasani N (2018). The diagnosis and management of nonalcoholic fatty liver disease: practice guidance from the American Association for the Study of Liver Diseases. Hepatology..

[4] Polyzos SA, Kechagias S, Tsochatzis EA (2021). Review article: non-alcoholic fatty liver disease and cardiovascular diseases: associations and treatment considerations. Aliment. Pharmacol. Ther..

[5] Loomba R (2020). Nonalcoholic fatty liver disease progression rates to cirrhosis and progression of cirrhosis to decompensation and mortality: a real world analysis of Medicare data. Aliment. Pharmacol. Ther..

[6] Wong VW-S, Adams LA, de Lédinghen V, Wong GL-H, Sookoian S (2018). Noninvasive biomarkers in NAFLD and NASH — current progress and future promise. Nat. Rev. Gastroenterol. Hepatol..

[7] Lazarus JV (2021). Defining comprehensive models of care for NAFLD. Nat. Rev. Gastroenterol. Hepatol..

[8] Lazarus JV (2022). The global NAFLD policy review and preparedness index: are countries ready to address this silent public health challenge?. J. Hepatol..

[9] Francque SM (2021). Non-alcoholic fatty liver disease: a patient guideline. JHEP. Rep..

[10] Lazarus JV (2022). Advancing the global public health agenda for NAFLD: a consensus statement. Nat. Rev. Gastroenterol. Hepatol..

[11] Li L (2016). Obesity is an independent risk factor for non-alcoholic fatty liver disease: evidence from a meta-analysis of 21 cohort studies. Obes. Rev..

[12] Mantovani A (2021). Non-alcoholic fatty liver disease and risk of incident diabetes mellitus: an updated meta-analysis of 501 022 adult individuals. Gut..

[13] Younossi ZM (2019). The global epidemiology of NAFLD and NASH in patients with type 2 diabetes: a systematic review and meta-analysis. J. Hepatol..

[14] Cusi K (2016). Treatment of patients with type 2 diabetes and non-alcoholic fatty liver disease: current approaches and future directions. Diabetologia..

[15] Francque SM, van der Graaff D, Kwanten WJ (2016). Non-alcoholic fatty liver disease and cardiovascular risk: pathophysiological mechanisms and implications. J. Hepatol..

[16] Dillon JF (2019). Intelligent liver function testing (iLFT): a trial of automated diagnosis and staging of liver disease in primary care. J. Hepatol..

[17] Kanwal F (2021). Clinical care pathway for the risk stratification and management of patients with nonalcoholic fatty liver disease. Gastroenterology..

[18] European Association for the Study of the Liver. (2021). EASL Clinical Practice Guidelines on non-invasive tests for evaluation of liver disease severity and prognosis − 2021 update. J. Hepatol..

[19] Targher G, Tilg H, Byrne CD (2021). Non-alcoholic fatty liver disease: a multisystem disease requiring a multidisciplinary and holistic approach. Lancet. Gastroenterol. Hepatol..

[20] Moolla A (2019). A multidisciplinary approach to the management of NAFLD is associated with improvement in markers of liver and cardio-metabolic health. Frontline. Gastroenterol..

[21] Haigh L (2019). Barriers and facilitators to mediterranean diet adoption by patients with nonalcoholic fatty liver disease in Northern Europe. Clin. Gastroenterol. Hepatol..

[22] Mazzotti A (2018). An internet-based approach for lifestyle changes in patients with NAFLD: two-year effects on weight loss and surrogate markers. J Hepatol..

[23] Vilar-Gomez E (2015). Weight loss through lifestyle modification significantly reduces features of nonalcoholic steatohepatitis. Gastroenterology..

[24] Aminian A (2021). Association of bariatric surgery with major adverse liver and cardiovascular outcomes in patients with biopsy-proven nonalcoholic steatohepatitis. JAMA..

[25] Pan CS, Stanley TL (2020). Effect of weight loss medications on hepatic steatosis and steatohepatitis: a systematic review. Front. Endocrinol..

[26] Rubino DM (2022). Effect of weekly subcutaneous semaglutide vs daily liraglutide on body weight in adults with overweight or obesity without diabetes: the STEP 8 randomized clinical trial. JAMA..

